# Potential Use of Sofosbuvir in the Prophylaxis for Rabies

**DOI:** 10.3389/fphar.2020.00472

**Published:** 2020-04-08

**Authors:** Sandra E. Reznik, Amit K. Tiwari, Charles R. Ashby

**Affiliations:** ^1^Department of Pharmaceutical Sciences, College of Pharmacy and Health Sciences, St. John’s University, Queens, NY, United States; ^2^Departments of Pathology and Obstetrics and Gynecology and Women’s Health, Montefiore Medical Center, Albert Einstein College of Medicine, Bronx, NY, United States; ^3^Department of Pharmacology and Experimental Therapeutics, College of Pharmacy and Pharmaceutical Sciences, University of Toledo, Toledo, OH, United States

**Keywords:** sofosbuvir, rabies, post-exposure prophylaxis, RNA-dependent RNA polymerase, virus, zoonotic virus

It is estimated that at least 59,000 people succumb to rabies each year, primarily in the rural areas of Asia and Africa (Hampson et al., 2015). These figures are widely assumed to be underestimated as number of rabies cases/year are thought to be higher. Once the clinical manifestations of rabies occur, the fatality rate is near 100% and rabies has the highest mortality rate of any known infectious disease. The rabies virus is a non-segmented, negative-stranded virus of the order Mononegavirales, family Rhabdoviridae, and genus *Lyssavirus* (Rupprecht and Gibbons, 2004). Rabies is a zoonotic viral disease that is transmitted by mammals and the primary sylvatic reservoir for rabies are species of the orders Chiroptera and Carnivora (Hatz et al., 2012). However, the primary vector for human rabies worldwide are canines (World Health, 2013). Rabies is typically transmitted to humans through a bite or scratch from a rabid animal, where infected saliva penetrates the skin or muscle or comes in contact with mucous membranes or open wounds (Nigg and Walker, 2009). In humans, rabies can be contracted in the absence of a bite or scratch from an infected animal, although the incidence is extremely rare (Nigg and Walker, 2009).

Currently, there are only seven well documented cases where humans have survived rabies (Nigg and Walker, 2009). Notably, six of these individuals received rabies vaccines prior to the onset of illness, suggesting that disease severity may have been mitigated by the vaccinations (Nigg and Walker, 2009). In 2004, a 15-year old girl from Wisconsin who was bitten on the hand by a bat survived rabies after using a therapeutic approach known as the Milwaukee protocol (Willoughby et al., 2005). However, to date, there have been at least 31 documented patients that did not survive following treatment with the Milwaukee protocol (Zeiler and Jackson, 2016). Furthermore, in its current form, only ketamine and midazolam remain as part of the protocol, thereby making it similar to standard intensive care procedures used around the world (Wilde and Hemachudha, 2015). Recently, it has been opined that the Milwaukee protocol should be abandoned (Zeiler and Jackson, 2016).

Following a recognized exposure to rabies, post-exposure prophylaxis (PEP) effectively prevents the onset of symptoms during the incubation period. Rabies PEP for people with WHO category II or III exposure involves: 1) immediate cleansing of the wound with alkaline soap and water for 15 min; 2) immediate passive immunization with human rabies immunoglobulin (HRIG), with most of the dose being infiltrated into the wound area; 3) active immunization with four (Zagreb regimen) or five (Essen regimen) for immunosuppressed patients, 1 ml-doses of a rabies vaccine given intramuscularly (Nigg and Walker, 2009; Hatz et al., 2012); or 4) intradermal or Thai Red Cross regimen (where approved by national health authorities, 0.1 ml per dose), given at two different lymphatic drainage sites (Nigg and Walker, 2009). However, there are certain issues related to rabies PEP. For example, in developing nations, where most of the cases of rabies occur, the use of HRIG is restricted by the cost (Jentes et al., 2013). Furthermore, the immune response to rabies vaccine may be inadequate in certain immune-compromised patients (Kopel et al., 2012). There is a limited supply of HRIG and it must be kept under refrigeration (2–8°C) (Imogam-Information, 2005). Unfortunately, many individuals in developing nations who have been exposed to a rabid animal will not be treated with HRIG (Both et al., 2012). Equine rabies immunoglobulin, which is less expensive, can be used, but it produces serum sickness (type III Gell-Coombs reaction) and precautions need to be taken regarding anaphylaxis (Jentes et al., 2013). Thus, at least in developing nations, which have the highest rabies burden, there is the need for effective treatments that could be used in place of HRIG for rabies PEP.

The replication of the rabies RNA genome occurs exclusively in the cytoplasm of the host cell and is catalyzed by the protein RNA-dependent RNA polymerase (RdRp or the L protein), which interacts with the nucleoprotein, N, and the non-enzymatic polymerase, cofactor P (Ivanov et al., 2011). The rabies RdRp is a 250 kD, multienzyme complex that in addition to synthesizing the RNA genome, also catalyzes the methylation and polyadenylation of the 5′- and 3′-end of the viral mRNA, respectively (Ivanov et al., 2011). The tertiary structure of all RdRp consists of three functional subdomains, designated the fingers, palm, and thumb that resemble the configuration of a cupped right hand (Te Velthuis, 2014; Jacome et al., 2015). The finger and thumb subdomains are primarily involved in the binding of the RNA primer and template (Jacome et al., 2015). In contrast, the catalytic subdomain in RdRp is located in the palm and it contains amino acids critical for positioning 1) the 3′-end of the RNA primer; 2) divalent cations; 3) template and 4) the incoming ribonucleotide triphosphate (rNTP) (Jacome et al., 2015). Six conserved structural domains have been shown to be present in all monomeric viral RdRp, known as subdomains A–F (Jacome et al., 2015). Subdomains A–E are in the palm and subdomains A and C are the most highly conserved (Te Velthuis, 2014; Jacome et al., 2015). The general mechanism of RNA polymerization by monomeric RdRp involves a highly conserved Asp in the A and C subdomains (for the rabies virus, Asn is the residue in motif C) in the palm subdomain that interact with two divalent metal ions (designated metals A and B) to facilitate a nucleophlic attack of the 5′-alpha-phosphate of the incoming rNTP with the 3′-OH of the RNA primer (Boehr et al., 2014). RdRp, following the binding of the rNTP, undergoes a structural conformation change that produces a catalytically closed conformation that ultimately catalyzes the formation of a phosphodiester bond between the incoming 5′-alpha-phosphate and the 3′-OH of the RNA primer terminus (Boehr et al., 2014; Shu and Gong, 2016). This nucleotidyl transfer reaction elongates the RNA template by one nucleotide until a complementary daughter RNA is formed. The RdRp represents a viable target for drug development as there is no homologous protein in human cells and inhibition of RdRp significantly decreases viral replication and viability. Previously, it has been reported that the L protein of the vesicular stomatitis virus (VSV) is homologous to rabies (as well as Ebola, measles and respiratory syncytial virus), with the same overall spatial configuration of the various domains and catalytic site residues (Liang et al., 2015). The modification of the 2′-OH of the RNA template and the rNTP position inhibits the activity of VSV RdRp *in vitro*, suggesting that 2′-modified nucleotides may be developed as antiviral drugs (Morin and Whelan, 2014). Interestingly, a number of 2′-modified nucleotides have been shown to inhibit RdRp activity in a number of viruses (Potisopon et al., 2017). It should be noted that the compound 6-fluro-3-hydroxy-2-pyrazinecarboxamide, also known as favipiravir, significantly 1) inhibits the replication of woodchuck and laboratory–adapted rabies strains in mouse neuroblastoma cells (neuro-2a) and 2) decreases morbidity and mortality in rabies–infected mice, at a dose of 300 mg/kg orally for 7 days (Yamada et al., 2016).

Based on the above information, we propose that sofosbuvir, a drug that is used to treat hepatitis C virus infections (genotypes 1–6), be tested as an alternative therapy for HRIG in rabies PEP. Sofosbuvir has α-fluorine and β-methyl substituents at the 2′-ribose position, which play an important role in its mechanism of action (Sofia, 2016). Indeed, sofosbuvir, a uridine analogue nucleotide phosphoramidate prodrug, is biotransformed *via* a series of enzymatic reactions to a triphosphate metabolite, 2′-fluoro-2-C-methyl-UTP (GS-461203) (Sofia, 2016). Subsequently, GS-461203 inhibits HCV RdRp activity, producing nonobligate RNA chain termination, which significantly decreases HCV replication and viral load in patients (Sofia, 2016).

Prior to clinical trials, we propose that sofosbuvir’s efficacy, along with the positive control, favipiravir, be determined: 1) *in vitro* against the most common circulating strains of rabies virus to ascertain if viral replication is significantly decreased by sofosbuvir; 2) *in vivo* in rabies-infected dogs, where its effect on virus neutralizing antibodies in plasma, brain viral load, and morbidity and mortality would be assessed.

If the results from the above experiments yield significant results, sofosbuvir’s efficacy would be determined in humans. As the safety of sofosbuvir has not been tested in children, this could be accomplished by identifying individuals 18 years of age or older who have a laboratory-confirmed diagnosis of rabies, in the absence of clinical signs/symptoms, for a double-blind, randomized, controlled trial. A 400-mg dose of sofosbuvir would be administered orally once daily for 21 days to individuals in the test group, while HRIG would be administered in the control group. The main efficacy endpoint would be lack of clinical symptoms of rabies and rabies-negative sera and saliva samples. Also, all patients during and after the trial would receive the best available supportive care. Since the use of sofosbuvir alone is not recommended for the treatment of HCV patients, all participants would be screened for HCV.

Safety should not be a concern for sofosbuvir as 1) no dose-limiting adverse effects have been reported; 2) only about 1% of HCV patients receiving 400 mg of sofosbuvir for 12 weeks discontinued treatment (Keating and Vaidya, 2014). The most frequently reported adverse effects are headache, fatigue, nausea, dizziness, and abdominal pain (Keating and Vaidya, 2014). However, if sofosbuvir elicits serious or intolerable adverse effects or increases mortality, treatment would be immediately withdrawn. Since the effect of sofosbuvir on fetal development and function is unknown, pregnant women would not be included in the current trial. Nonetheless, it should be noted that sofosbuvir, at concentrations significantly greater than that required for anti-HCV efficacy, does not produce mutagenesis, affect embryo-fetal development or impair fertility in animals (Sovaldi-Insert, 2015). In addition, *in vitro*, sofosbuvir has no toxic effects in a variety of normal human cell lines and is not toxic to mitochondria (Feng et al., 2016). Presently, there are no reports of sofosbuvir interfering with the efficacy of live or attenuated vaccines.

Sofosbuvir has a number of pharmacokinetic characteristics that make its use suitable in a wide range of patients. For example, it has good oral bioavailability (80%) and can be taken with or without food (Kirby et al., 2015). Sofosbuvir can be used in patients with mild-moderate renal and severe liver impairment (Kirby et al., 2015). It also has a relatively wide volume of distribution and its primary metabolite, GS-331007, is not toxic, does not accumulate to a significant extent after multiple dosing and lacks efficacy (Kirby et al., 2015). Given that sofosbuvir’s metabolism does not involve cytochrome P450 (CYP450) or uridine 5′-diphospho-glucuronosyltransferase (UDPGT) enzymes, drugs that induce or inhibit these enzymes will not affect its plasma or tissue levels (Kirby et al., 2015). Also, sofosbuvir and GS-331007 do not significantly inhibit or induce CYP450 or UDGPT enzymes; thus, they should not alter the efficacy or toxicity of drugs that are substrates for these enzymes (Kirby et al., 2015). Sofosbuvir is a substrate for the intestinal ATP cassette binding (ABC) transporters p-glycoprotein and breast cancer resistant protein; therefore, drugs that inhibit and induce these efflux proteins would increase and decrease, respectively, the plasma levels of sofosbuvir (Kirby et al., 2015). Cyclosporine, tacrolimus, and methadone increase, whereas rifampin and carbamazepine decrease, the plasma levels of sofosbuvir (Garrison et al., 2018). Sofosbuvir increases the plasma levels of darunavir + ritonavir and norgestrel (Garrison et al., 2018). Finally, current pharmacokinetic data suggest that there should be minimal drug-drug interactions between sofosbuvir and a number of common clinically used drugs.

The cost of sofosbuvir is an important issue regarding its use for rabies PEP. However, the cost of generic sofosbuvir in India, a country that bears a large rabies burden, ranges from $161 to $312 for 28 tablets (Iyengar et al., 2016). Thus, a 3-week PEP regimen of sofosbuvir, as proposed in this paper, would cost 120 to 234 U.S. dollars. Furthermore, Hill et al., based on the fact that sofosbuvir is closely analogous to the API cost of synthesizing stavudine, estimated that the cost for a 12-week regimen of sofosbuvir would be 68–136 U.S. dollars, or 17–34 U.S. dollars for a 3-week PEP regimen (Hill et al., 2014). These aforementioned costs are significantly lower than the price of HRIG alone.

In summary, we predict that sofosbuvir, an RdRp inhibitor currently used to treat HCV, would also inhibit the rabies RdRp and therefore be efficacious in rabies PEP. Sofosbuvir is well tolerated and has suitable pharmacokinetic properties. If preclinical trials are successful, we propose that sofosbuvir be tested for its efficacy against rabies virus in double–blind, randomized controlled trials. Although the nominal ex-factory cost of sofosbuvir is high, it has been hypothesized that the cost of sofosbuvir can be affordable, making it accessible in the developing world where most cases occur.

## Author Contributions

SR, CA, and AT discussed and agreed on the presented opinion. CA wrote the draft and SR and AT contributed to a portion of the draft. CA, AT, and SR proofed the article.

## Conflict of Interest

The authors declare that the research was conducted in the absence of any commercial or financial relationships that could be construed as a potential conflict of interest.
